# Alpha-lipoic acid preserves skeletal muscle mass in type 2 diabetic OLETF rats

**DOI:** 10.1186/s12986-018-0302-y

**Published:** 2018-09-29

**Authors:** Oak-Kee Hong, Jang-Won Son, Hyuk-Sang Kwon, Seong-Su Lee, Sung-Rae Kim, Soon Jib Yoo

**Affiliations:** 1 Department of Internal Medicine, College of Medicine, The Catholic University of Korea, 222, Banpo-daro, Seocho-gu, Seoul, 06591 Republic of Korea; 2 Division of Endocrinology and Metabolism, Department of Internal Medicine, Bucheon St. Mary’s Hospital, College of Medicine, The Catholic University of Korea, 327, Sosa-ro, Wonmi-gu, Bucheon-si, Gyeonggi-do 14647 Republic of Korea; 3 Division of Endocrinology and Metabolism, Department of Internal Medicine, Yeouido St. Mary’s Hospital, College of Medicine, The Catholic University of Korea, 10, 63-ro, Yeongdeungpo-gu, Seoul, 07345 Republic of Korea

**Keywords:** Alpha-lipoic acid, Diabetic rat, Diabetes mellitus, Skeletal muscle, Muscle mass

## Abstract

**Background:**

Increased oxidative stress and impaired antioxidant defense are important mechanisms in the pathogenesis of diabetic myopathy. Alpha-lipoic acid (ALA) has been indicated as a weight-loss treatment in rodents and humans, but studies are limited. In the present study, we aimed to determine the influence of ALA, a potent biological antioxidant, on metabolic and growth processes in diabetic rat skeletal muscle.

**Methods:**

Male 25-week-old type 2 diabetic rats (OLETF) were randomly divided into two groups, a control group (OLETF-C) and an ALA-treated group (OLETF-ALA) supplemented with 100 mg/kg ALA for 8 weeks. Age-matched, healthy, nondiabetic LETO (LETO-C) rats were used as controls.

**Results:**

At 32 weeks of age, body weight was decreased by 6.8%, and the areas under the curve of IP-GTT, fasting glucose, and insulin were less in OLETF-ALA rats compared with OLETF-C rats. ALA significantly preserved muscle mass and enhanced muscle fiber cross-sectional area and fiber frequency percentage in the skeletal muscle of OLETF rats. Although the activation of myoD, myogenin, and myostatin in gastrocnemius muscle was significantly inhibited in OLETF-ALA rats relative to OLETF-C rats, there were no differences in the expression levels of muscle atrogin-1 and MuRF1 between the two groups. ALA treatment significantly increased the levels of phosphorylated 5′-AMPK, SIRT1, and PGC-1α, as well as the levels of phosphorylated AKT, mTOR, and p70S6 kinase in OLETF-ALA rats compared with OLETF-C rats. In contrast, the levels of phosphorylated p38 MAPK, IRS-1, and FOXO1 were decreased in OLETF-ALA rats compared with OLETF-C rats.

**Conclusions:**

ALA treatment preserved mass in the gastrocnemius muscles of OLETF rats. ALA significantly upregulated the AMPK/SIRT1/PGC-1α and AKT/mTOR/p70S6K signaling pathways in OLETF rat skeletal muscle. Therefore, ALA may be a potential therapeutic intervention for skeletal muscle loss in animal models of insulin resistance.

## Background

Skeletal muscle is the most abundant tissue in the vertebrate body, and one of the most dynamic and plastic tissues in the human body [1]. In humans, skeletal muscle comprises approximately 40% of total body weight and contains 50–75% of all body proteins [1]. The maintenance of skeletal muscle mass plays an important role in disease prevention and quality of life [2–5]. Muscle mass, which can change quite rapidly, is the result of a dynamic balance between protein synthesis and degradation. Both processes are sensitive to factors such as nutritional status, hormonal balance, physical activity/exercise, and injury or disease [2–5].

Alpha-lipoic acid (ALA) is a sulfur-containing coenzyme involved in mitochondrial dehydrogenase reactions leading to adenosine triphosphate (ATP) formation [6]. ALA, along with its major metabolite dihydrolipoic acid (DHLA), is a potent antioxidant via its scavenging of oxygen free radicals, redox interactions with other antioxidants, and inhibition of lipid peroxidation [6]. The beneficial effects of ALA in diabetes have long been recognized, and are mainly attributed to its involvement in glycemic control, increasing antioxidant markers, and reducing body weight [7–9]. More recently, ALA was deemed safe and effective in the treatment of diabetic end-organ complications, such as diabetic retinopathy, diabetic neuropathy, diabetic cardiomyopathy, and leg wound healing [10, 11].

ALA has direct effects on skeletal muscle, increasing resting energy expenditure, insulin sensitivity, and glucose uptake [12]. These effects are primarily mediated through the activation of AMP-activated protein kinase (AMPK), which regulates mitochondrial biogenesis through peroxisome proliferator-activated receptor γ coactivator 1α (PGC-1α) in the skeletal muscles of aged mice [13, 14]. Thus, activation of AMPK by ALA may lead to a lower accumulation of intramyocellular lipids via the increased oxidation of long-chain fatty acids, and may improve whole-body insulin sensitivity [10, 13, 14]. ALA also promotes protein synthesis through the protein kinase B (AKT)/mammalian target of rapamycin (mTOR) signaling pathway in C2C12 myotubes [15] as well as in obese Zucker rats [16]. However, the role of ALA in attenuating diabetic muscle mass has not been extensively investigated.

Research focusing on mechanism in which ALA regulates skeletal muscle mass of diabetes animal modes in vivo is limited. The primary aim of the present study was to assess whether ALA treatment impact increasing skeletal muscle mass in an animal model of type 2 diabetes. Additionally, we investigated the potential signaling pathways underlying ALA modulation of AMPK/sirtuin 1 (SIRT1)/PGC-1α and AKT/mTOR/p70S6K signaling, and other protein regeneration and degradation factors.

## Methods

### Animals

Five-week-old male Otsuka Long-Evans Tokushima Fatty (OLETF) rats and Long-Evans Tokushima Otsuka **(**LETO) rats were obtained from the Otsuka Pharmaceutical Tokushima Research Institute (Tokushima, Japan) and maintained in a temperature (23 ± 3 °C) and humidity (55 ± 5%) controlled room with a 12 h:12 h light:dark cycle. Animals were provided a standard rat chow (Samyangsa, Seoul, Korea) and water ad libitum.

### ALA treatments

Twenty-five-week-old OLETF and LETO rats, weighing between 450 g and 550 g, were provided with water supplemented with 0 mg/Kg (*n* = 10, OLETF-C, OLETF control treatment group) or 100 mg/Kg/day in drinking water (*n* = 10, OLETF-ALA, OLETF ALA treatment group, Bukwang R&D, Seoul, Korea) for 8 weeks. Age-matched, healthy nondiabetic Long-Evans Tokushima Otsuka (LETO) rats (*n* = 10) were used as controls. Body weight and fed plasma glucose were monitored weekly. All rats were fed standard chow and all animals were pair-fed to ensure similar feed and calorie intake between the OLETF-C and OLETF-ALA groups. Water intake were monitored daily. The end of the 8 week study, the gastrocnemius muscle was excised from the right and left leg. Epididymal, retroperitoneal fat pads, liver, heart and pancreas weighted for morphometric assessment and expressed as tissue mass g per g body weight. The sample stored at − 80 °C until analysis.

### Intraperitoneal glucose tolerance tests (IP-GTT)

After 8 weeks of ALA treatment, all experimental rats were fasted overnight, and intraperitoneal glucose tolerance tests (25% glucose solution, 2 g/kg) were performed. Blood glucose levels were measured using a glucose oxidase method with one-touch test strips (Accuchek, Roche Diagnostics, Mannheim, Germany).

### Biochemical analysis

Serum concentrations of fasting glucose were determined with an automatic blood chemical analyser (Cobas 8000, Roche Diagnostics). Insulin was assayed using the ELISA Kits (Merk, Damstadt, Germany) according to the manufacturer’s instruction. All the reagents and samples were stored at room temperature before conducting the experiment.

### Histological analysis

Cross sections (10 μm^2^ thick) were cut from the middle portion of the gastrocnemius muscle with paraffin block. The sections were stained with hematoxylin and eosin (HE). At least 5 sections were taken from each sample and at least 10 microscopic fields were examined at × 100 magnification by using Olympus AX70 microscope (Olympus, Tokyo, Japan). The cross-sectional size of muscle fibers was evaluated by measuring the minimal diameter and the cross-sectional areas (CSA) of muscle fibers with image analysis software (Image J 1.49) and averaged to obtain the mean value in each rat. The frozen sections of epididymal and retroperitoneal fat were stained with HE.

### Western blot

Skeletal muscle tissues protein was extracted with ice-cold lysis buffer. Immunoblots were obtained using antibodies against the following proteins: Atrogin-1/Fbx32, muscle-specific- RING-finger protein 1 (MuRF1), PGC-1α (all from Abcam, Cambridge, UK), GADPH (Abclon, Seoul, Korea), phosphorylated-5’-AMP-activated protein kinase (AMPK (Thy^172)^), AMPK, phosphorylated- p38 MAP kinase (p38 MAPK), p38 MAPK, phosphorylated-AKT (Ser^473^), AKT, phosphorylated-forkhead box O 1 (Ser^256^) (FOXO1), FOXO1, phosphorylated-mTOR (Ser^2448^), mTOR, phosphorylated-p70S6 kinase (Thr^389^), p70S6 kinase (Cell Signaling Technology Inc., Danvers, MA), and myogenin determination gene D (myoD), myogenin, myostatin, sirtuin 1 (SIRT1) (Santa Cruz Biotechnology, Inc., Dallas, TX), phosphorylated-insulin receptor substrate-1 (Ser^307^), IRS-1 (Merk). Detection was achieved using an SuperSignal West Pico Chemiluminescent Substrate (Thermo Fisher Scientific, Seoul, Korea). Quantitation of Western blots by densitometry was performed with GeneTools 4.03 software (SynGene, Cambridge, England).

### Statistical analysis

Data are expressed as means ± SEM. Differences between groups were evaluated using the SimgaPlot 13 program (Systat Software, Inc., San Jose, CA). Data were tested for normality (Shapiro-Wilk test) and equality of variance (Kruskal-Wallis test). When both conditions were met, an unpaired t-test or one-way analysis of variance was performed, as appropriate. When the normality or equality of variance conditions was not met, the variables were analyzed using the Brown-Forsythe test, Mann-Whiteney U-test as appropriate. The Turkey, Bonferroni, Dunn’s or Holm-Sidak post hoc test was used as needed. The significance level was set at *P* < 0.05.

## Results

### ALA prevents weight gain and improves glucose control

**Fig. 1:**
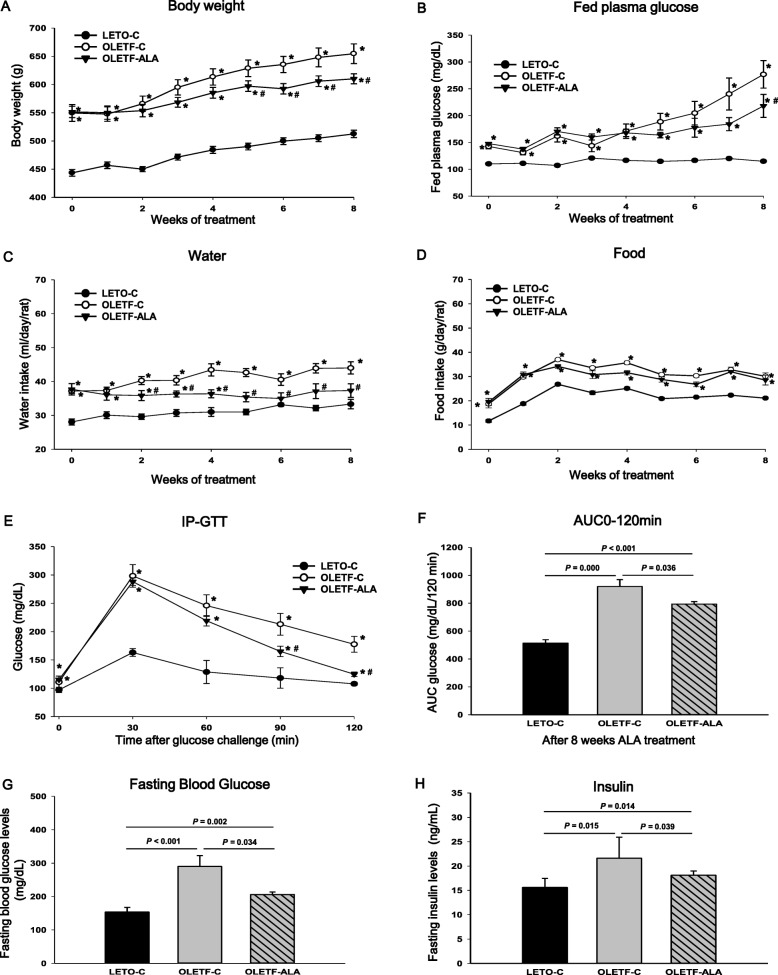
ALA –treated OLETF rats had significantly lower final body weight, and glucose and insulin than the control OLETF rats. **a** Body weight was measured for 8 weeks of ALA treatment. **b** Fed plasma glucose, **c** water intake, **d** food intake was measured for 8 week of ALA treatment. The IP-GTT analyses were performed after 8 weeks of ALA treatment. **e** IP-GTT and **f** AUC of IP-GTT were assessed using blood glucose levels following an intraperitoneal injection of a 25% glucose solution (2 g/kg) after the rats had been fasted for 18 h. The **g** fasting blood glucose, **h** fasting insulin was measured at 8 weeks of ALA treatment. Data presented as mean ± SEM (LETO-C: *n* = 10; OLETF-C: *n* = 10; OLETF-ALA: *n* = 10). *: *P* < 0.05 vs. LETO-C. #: *P* < 0.05 vs. OLETF-C

The increase in the weight of the OLETF rats was greater than that of the LETO-C rats, regardless of ALA administration (Fig. 1a). As expected, body weight was significantly increased in OLETF-C rats compared with OLETF-ALA rats at the end of the treatment period (653.3 ± 7.9 g vs. 611.8 ± 7.9 g, respectively; *P* < 0.05), as ALA prevented body weight increase in OLETF rats (Fig. 1a). OLETF-C rats also displayed marked and significant increases in fed plasma glucose levels relative to OLETF-ALA rats (*P* < 0.05, Fig. 1b), although daily food intake for the duration of the feeding trial did not differ between the two groups (Fig. 1c and d). After 8 weeks of ALA treatment, fasted OLETF-ALA rats had significantly lower blood glucose levels at 90 and 120 min post-glucose challenge than fasted OLETF-C rats (*P* < 0.05, Fig. 1e). The AUC of IP-GTT of the OLETF-ALA rats exhibited a significant decrease compared with age-matched untreated OLETF-C rats (920.0 ± 50.0 vs. 794.1 ± 17.9, respectively; *P* < 0.05, Fig. 1f). Serum fasting glucose also were remarkably decreased by 0.3-fold compared with OLETF-C rats (206.0 ± 7.3 vs. 290.0 ± 32.5, *P* < 0.05, Fig. 1g). Furthermore fasting insulin were significantly decreased by 0.3-fold compared with OLETF-C rats (17.7 ± 1.2 vs. 23.6 ± 2.3, *P* < 0.05, Fig. 1f). Diabetic rats (OLETF-C and OLKETF-ALA) had significantly greater fasting glucose level (Fig. 1b, e, f and g), insulin level (Fig. 1h) and food intake (Fig. 1c and d) than age-matched nondiabetic rats (LETO-C) (*P* < 0.05).

### ALA attenuates muscle mass and morphological changes in muscle tissue

**Fig. 2:**
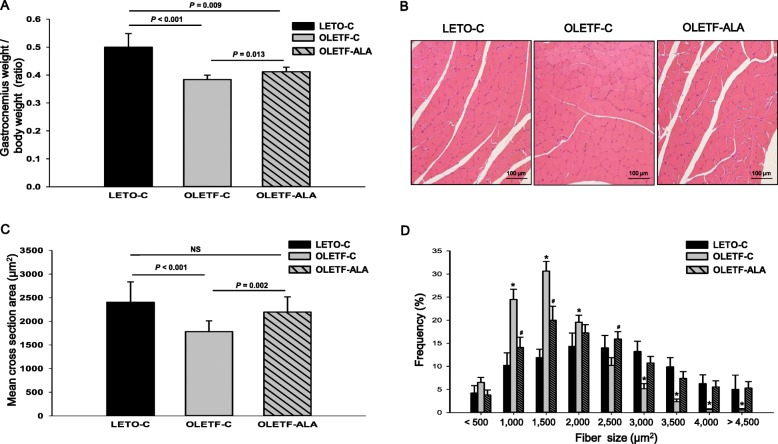
ALA increased body proportion of muscle mass, and mean cross section area in OLETF rats. The end of the 8 week study, gastronecmius weighted for morphometric assessment and expressed as tissue mass g per g body weight. **a** Relative muscle mass **b** representative macrographs of hematoxylin and eosin (H&E) stained gastrocnemius muscle sections in LETO and OLETF rats (X100). Scale bar = 100 μm. Fiber (**c**) mean cross-sectional area (CSA) and **d** distribution in LETO and OLETF gastrocnemius muscle. Data presented as mean ± SEM (*n* = 5–6 rats per group). *: *P* < 0.05 vs. LETO-C. #: *P* < 0.05 vs. OLETF-C

We examined the effects of ALA on gastrocnemius muscle weight and morphology, and also examined muscle size in terms of fiber diameter. Gastrocnemius muscle weights were measured at the time of sacrifice, and the gastrocnemius muscle wet weight/body weight ratio (ww/bw) was calculated for each group. OLETF relative muscle weights were lower than those of LETO-C rats, also the muscle ww/bw was significantly lower in OLETF-C rats than in OLETF-ALA rats (*P* < 0.05, Fig. 2a). Histological analysis revealed no abnormal organization of muscle tissue (such as fibrosis) (Fig. 2b). The significant increase in relative muscle mass observed in OLETF-ALA rats (*n* = 83–132 fibers from each of 6 rats: 2,197 ± 83 μm^2^) was due to a marked increase in muscle fiber size, which was particularly evident in the fiber diameter of the cross-sectional area in OLETF-ALA rats compared with OLETF-C rats (*n* = 88–173 fibers from each of 5 rats: 1,784 ± 57 μm^2^) (*P* < 0.05, Fig. 2c) and fiber frequency curve of OLETF-ALA shifted towards larger fibers (> 2,000 μm^2^) compared with OLETF-C (*P* < 0.05, Fig. 2d). The average fiber diameter in the OLETF rats was smaller than that in the LETO control rats (LETO-C, *n* = 86–111 fibers from each of 5 rats: 2,405 ± 143 μm^2^).

### ALA attenuates the decrease in the epididymal fat weight/body weight ratio and epididymal and retroperitoneal fat morphology

**Fig. 3:**
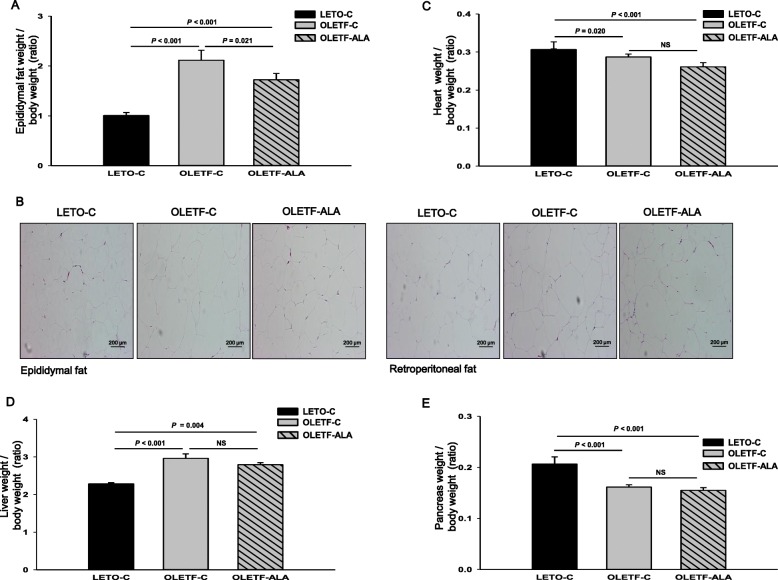
ALA decreased body portion of fat mass of OLETF rats. The end of the 8 week study, **a** epididymal fat pads, **c** heart **d** liver and **e** pancreas weighted for morphometric assessment and expressed as tissue mass g per g body weight. **b** The representative macrographs of hematoxylin and eosin (H&E) stained epididymal and retroperitoneal fat in LETO and OLETF rats (X200). Scale bar = 200 μm. Data presented as mean ± SEM (*n* = 10 rats per group). *: *P* < 0.05 vs. LETO-C. #: *P* < 0.05 vs. OLETF-C

Treatment of OLETF rats with ALA significantly reduced epididymal fat weight gain by approximately 20% (*p* < 0.5, Fig. 3a). Nevertheless, we did not measure retroperitoneal fat weight, or epididymal or retroperitoneal fat morphology following treatment with ALA (Fig. 3b). The heart, liver, and pancreas weights of OLETF-ALA rats were less than those of OLETF-C rats, although the difference did not reach statistical significance (Fig. 3c–e).

### ALA suppresses myogenic regulatory factors (MRFs) in muscle tissue

**Fig. 4:**
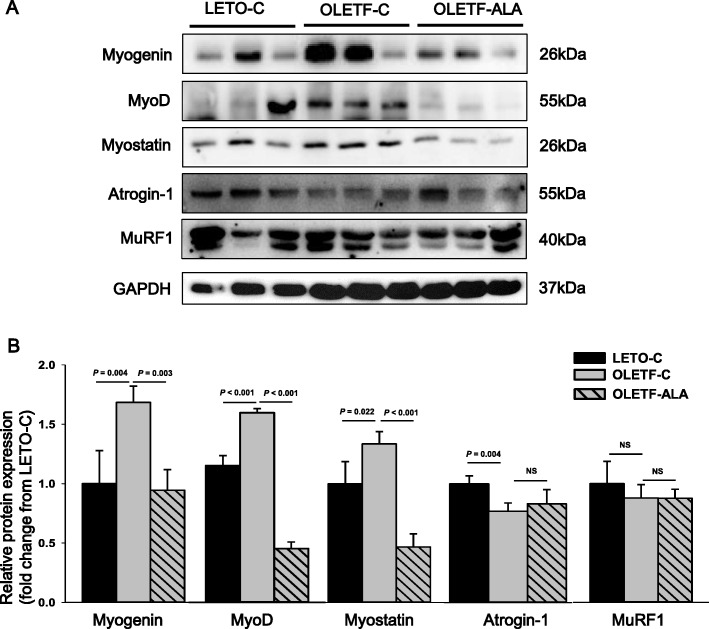
ALA was downregulated protein expression levels of MyoD, myogenin, and myostatin in the skeletal muscles of OLETF rats. MyoD, myogenin, myostatin, atrogin-1, and MuRF1 expression levels in rat skeletal muscle were determined after 8 weeks of ALA treatment using **a** western blot analyses, **b** scanning densitometry. Band intensities were normalized to GAPDH. Data presented as mean ± SEM (*n* = 6 rats per group)

To further explore the significance of ALA as a regulator of skeletal muscle growth, we analyzed the myogenin-regulating factors (MRFs) myogenin determination gene D (MyoD) and myogenin, and the atrogenic factors myostatin, atrogin-1, and muscle RING-finger protein-1 (MuRF1) in skeletal muscles of diabetic rats. Although atrogin-1 and MuRF1 of the LETO-C rats was shown to increase compared to that in OLETF rats, myogenin, MyoD, and myostatin of LETO-C rats was decreased. ALA treatment significantly downregulated MRFs in OLETF rats compared with OLETF-C rats (*P* < 0.05, Fig. 4a and b). MRF protein levels were higher in OLETF-C rats than in age-matched LETO-C rats (*P* < 0.05, Fig. 4a and b). Although the action of myostatin was significantly reduced in OLETF-ALA rats compared with OLETF-C rats (*P* < 0.05), the levels of atrogin-1 and MuRF1 did not differ between the two groups (Fig. 4a and b).

### ALA activates the AMPK/SIRT1/PGC-1α signaling pathway in muscle tissue

**Fig. 5:**
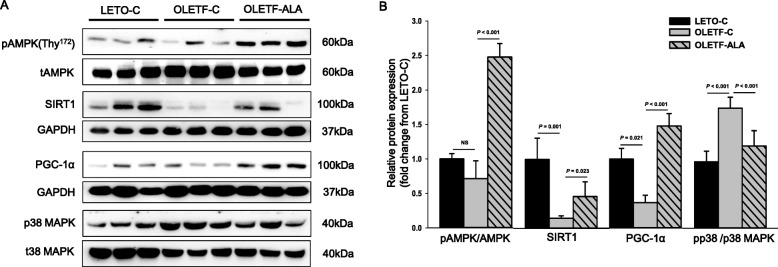
ALA upregulated protein expression levels of AMPK, SIRT1, and PGC-1α in the skeletal muscles of OLETF rats. The phosphorylation of AMPK and p38 MAPK, and AMPK, SIRT1, PGC-1α, p38 MAPK expression levels in rat skeletal muscle were determined after 8 weeks of ALA treatment using **a** western blot analyses, **b** scanning densitometry. Band intensities of SIRT1 and PGC-1α were normalized to GAPDH. Data presented as mean ± SEM (*n* = 6 rats per group)

Figure 5 shows a representative western blot of SIRT1, AMPK, p38 mitogen-activated protein kinase (MAPK), and PGC-1α levels in skeletal muscle of LETO and OLETF rats. The SIRT1, phosphorylated AMPK, and PGC-1α protein levels in OLETF-C rats were lower than those in age-matched LETO-C rats except the phosphorylation of p38 MAPK (*P* < 0.05, Fig. 5). The levels of phosphorylated AMPK and PGC-1α were significantly higher in OLETF-ALA rats than in OLETF-C rats (*P* < 0.05, Fig. 5a and b). Moreover, SIRT1 protein levels were significantly higher in OLETF-ALA rats than in OLETF-C rats (*P* < 0.05, Fig. 5a and b). The activation of p38 MAPK in OLETF-ALA rats was lower than in OLETF-C rats (*P* < 0.05, Figs. 5a and 5b).

### ALA activates the AKT/mTOR/p70S6K signaling pathway in muscle tissue

**Fig. 6:**
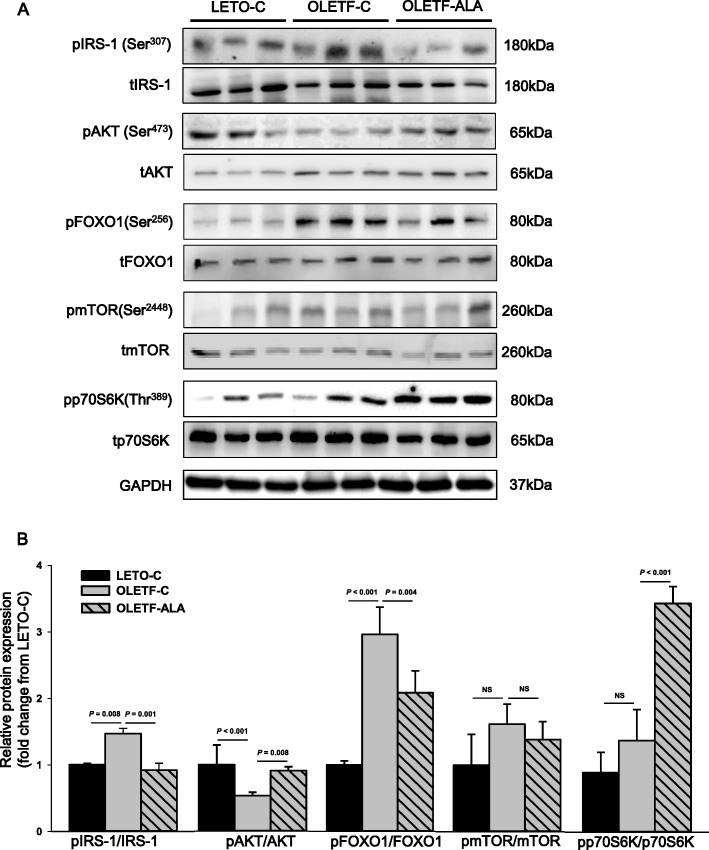
ALA was upregulated protein expression levels of AKT and p70S6K in the skeletal muscles of OLETF rats. The phosphorylation of AKT, FOXO1, mTOR, p70S6K and AKT, FOXO1, mTOR, p706SK expression levels in rat skeletal muscle were determined after 8 weeks of ALA treatment using **a** western blot analyses, **b** scanning densitometry. Data presented as mean ± SEM (*n* = 6 rats per group)

To investigate ALA modulation of AKT/mTOR signaling pathways, we analyzed IRS-1, AKT, forkhead box O1 (FOXO1), mTOR, and p70S6K expression levels by western blotting. The phosphorylation of IRS-1, FOXO1, mTOR, and p70S6K of OLETF rats was shown to increase compared with that in the LETO-C rats except the phosphorylation of AKT. The activation of IRS-1 in OLETF-ALA rats was lower than in OLETF-C rats (*P* < 0.05, Fig. 6a and b). ALA increased the phosphorylation of AKT in OLETF-ALA rats relative to OLETF-C rats (*P* < 0.05, Fig. 6a and b). Although the phosphorylation of FOXO1 was significantly decreased in OLETF-ALA rats (*P* < 0.05), phosphorylated mTOR levels did not differ between the two groups (Fig. 6a and b). However, p70S6K phosphorylation was higher in ALA-treated rats than in controls (*P* < 0.05, Figs. 6a and b).

## Discussion

Previous studies have shown that ALA suppresses protein synthesis in skeletal muscle by downregulating the mTOR signaling pathway [16] and enhancing AMPK-PGC-1α-mediated mitochondrial biogenesis in the skeletal muscle of aged mice [13]. However, the effects of ALA on the metabolic and growth processes of diabetic skeletal muscle remain unclear. Here, we discuss several important pathways and factors, including the AMPK-SIRT1-PGC-1α pathway (involved in energy metabolism), MRFs (involved in muscle regeneration), atrogenic factors (involved in muscle degradation), and the AKT-mTOR pathway (involved in protein synthesis), based mostly on results from animal studies.

Importantly, the present study demonstrated that ALA prevented body weight increase in a type 2 diabetic animal model using OLETF rats, as has also been shown in Zucker diabetic fatty (ZDF) rats [16, 17]. This is in agreement with a recent study demonstrating that ALA reduces body weight and regulates triglycerides in obese patients with diabetes mellitus [7–9]. However, relative muscle mass as a percentage of body composition, as well as cross-sectional area, actually increased in the ALA-treated OLETF rats (Fig. 2a, c, d). We also demonstrated an increase in skeletal muscle weight (gastrocnemius) in the change of total body weight, although other measures of body fat, such as the size of different fat deposits (epididymal and retroperitoneal fat), may have changed (Fig. 3). Our results also showed that ALA administration blunted body weight gain, which was associated with smaller fat size in the white adipocytes of ALA-treated OLETF rats (Fig. 3a, b). An increase in muscle mass is associated with reduced insulin resistance, which can be estimated by the homeostasis model assessment of insulin resistance [2–5]. In this study, ALA-treated OLETF rats exhibited improved fasting glucose levels and insulin sensitivity.

It is unclear whether ALA-mediated loss of body mass resulted from increased rates of protein degradation and/or decreased rates of protein synthesis. We could not determine whether the increased muscle mass was due to muscle hypertrophy or hyperplasia. According to Park et al. [18], gastrocnemius mass and myofiber cross-sectional area were greater with β-hydroxy-β-methylbutyrate (HMβ) treatment in the presence of a calorie-restricted diet compared to caloric restriction alone. These findings were also associated with reduced levels of the ubiquitin ligase, MAFbx, indicating a reduction in protein degradation. Abdai et al. [14] reported that antioxidant supplementation specifically suppressed p38 MAPK activation following acute exercise. Malavaki et al. [19] found that the atrogin-1/MAFbx gene is a downstream target of p38 MAPK signaling, and hindlimb suspension or immobilization in rats resulted in elevated p38 MAPK activity in the soleus muscle. PGC-1α acts as an upstream regulator of atrogin-1 and MuRF-1. PGC-1α was induced by feeding nutrient-enriched food (ALA, ALCAR, and HT) to rats [20]. Furthermore, p38 MAPK also induces the expression and activation of PGC-1α, which promotes skeletal muscle oxidation [14, 21]. It is not clear whether ALA promotes PGC-1α transcription in skeletal muscle through activation of the p38 MAPK pathway.

Certain skeletal muscle growth and regeneration processes may be influenced by key myogenic factors. MRFs may be required to maintain muscle homeostasis and could play a role in muscle plasticity in response to both hypertrophic and atrophic stimuli [4, 5, 22, 23]. Myogenin, as an essential regulator of myogenesis, promotes muscle atrophy upon denervation by directly activating the expression of atrogin-1 and MuRF1 [4, 5, 23–25]. Myostatin decreases the expression of these atrophy markers in differentiated myotubes, as well as myoD and myogenin, which are normally upregulated during differentiation [23, 24]. Myostatin also acts to block muscle size and the AKT pathway. Thus, by blocking myostatin, PI3K/AKT activation stimulates differentiation and protein synthesis via this mechanism [4, 23–25]. AKT induces protein synthesis and blocks the transcriptional upregulation of key mediators of skeletal muscle atrophy, such as atrogin-1 and MuRF1. FOXO proteins, belonging to the Forkhead family of transcription factors, have also been linked to muscle atrophy. FOXOs have been implicated in the regulation of skeletal muscle mass. Phosphorylated AKT represses FOXO, inhibiting protein degradation [2–5, 23–25] and regulating the expression of genes encoding atrogin-1/MAFbx and MuRF1 ligases [2–5, 23–25]. Increased skeletal muscle mass by ALA could be, at least in part, associated with inhibition of the activities of atrogin-1 and MuRF-1.

A number of studies have suggested that ALA can increase glucose clearance by modulating insulin action, which improves insulin sensitivity via glucose uptake in skeletal muscle [12–16]. All of the aforementioned actions resulting from ALA activation of AMPK result in decreased plasma glucose levels, increased insulin sensitivity [6, 11, 13–16], and likely in weight loss [7–9]. Indeed, these actions of ALA-AMPK may also increase energy expenditure by increasing the activity of the PGC-1α signaling pathway, which is responsible for mitochondrial biogenesis [13, 17]. Mitochondrial morphology and function may play key roles in the maintenance of muscle mass; thus, AMPK and PGC-1α may play fundamental roles in the regulation of skeletal muscle mass by controlling both protein synthesis and degradation [25, 26]. Activation of SIRT1 improves mitochondrial function, and the modulating effects of SIRT1 on energy homeostasis in skeletal muscle may require an upstream activation signal mediated through the energy sensor AMPK in response to resveratrol stimulation [27, 28]. Our results were similar to the effects of resveratrol on skeletal muscle. Resveratrol, a SIRT1 activator, protects against obesity and the development of insulin resistance [27]. Controversial reports suggest that AMPK activates PGC-1α either directly or indirectly by activating SIRT1. However, we showed significant reduction in the phosphorylation of AMPK, SIRT1, and PGC-1α in OLETF-C rats, while ALA treatment reversed these effects (Fig. 5), consistent with the results of Lee [29]. The tocotrienol-rich fraction (TRF) also prevented muscle atrophy associated with diabetes by regulating insulin signaling via the AMPK/SIRT1/PGC-1α pathways in type 2 diabetic mice [29]. Berberine treatment in aged rats improved muscle function by activating the AMPK/SIRT1/PGC-1α pathways [30]. The role of the AMPK/SIRT1/PGC-1α pathway in skeletal muscle is related to energy homeostasis under diabetic conditions and attenuates mitochondrial dysfunction [30–32]. SIRT1 is a key regulator of mitochondrial biogenesis through the deacetylation of PGC-1α in skeletal muscle cells [30]. Therefore, our studies suggest that the activation of AMPK by ALA increased the deacetylation activity of SIRT1 and enhanced PGC-1α.

In vitro, ALA induces protein synthesis in muscle through the PI3K/AKT pathway [15]. Insulin and insulin-like growth factors (IGFs), IGF-I and IGF-II, are mainly involved in muscle hypertrophy and play pivotal roles in growth, differentiation, survival, and various aspects of metabolism in a wide range of mammalian tissues [4, 23–25]. Modulation of IGF-1 signaling is required for the downregulation of IRS-1 phosphorylation, and the P3K/AKT/mTOR signaling pathway activates IRS-1 degradation [33]. Activated AKT, in turn, phosphorylates and activates a second kinase, mTOR [4, 16, 33, 34]. In disagreement with previous reports [13], our data showed that ALA induced IRS-1 phosphorylation and subsequent stimulation of the PI3K/AKT pathway, resulting in activation of the p70S6 pathway in skeletal muscle of OLETF rats. Although activation of mTOR is necessary and sufficient to induce IRS-1 degradation, our data suggest that the phosphorylation of mTOR did not differ between the two groups. Yoneyama et al. [33] reported that IRS-1-mTORC1 has a feedback loop that acts in response to temporal regulation of IGF-1 signaling. Obese and high-fat-diet-fed mice exhibit elevated mTORC1 signaling, which is believed to be due to overdriving feedback inhibition of IRS-1 by insulin resistance [33, 34]. However, future studies will be necessary to determine whether ALA directly affects IRS-1-mTORC1 in the insulin sensitivity state in muscle cell lines.

Because AMPK is a negative regulator of skeletal muscle hypertrophy, activated AMPK by ALA likely suppresses protein synthesis in rat skeletal muscle by inhibiting mTORC1 activity [16]. SIRT1 has been reported to increase insulin signaling and IGF-I expression in muscle in insulin-resistant states [35, 36]. SIRT1 appears to be fundamental to skeletal muscle cell survival, as it regulates the AKT/mTOR signaling pathway linked to muscle growth [34]. Wang et al. [37] reported that the activation of SIRT1 by resveratrol restored AKT/mTOR/S6K and reduced FOXO1 in TNF-α- stimulated skeletal muscle cells. mTOR and SIRT1 pathways increase organism life/health-spans and are fundamental in protein synthesis, growth, differentiation, and survival in skeletal muscle in old age [35]. Many studies have shown how SIRT1 is associated with the AMPK and mTOR pathways. Furthermore, a significant increase in SIRT1 is particularly important for achieving more significant physiological and pathologic activities of muscle [26]. Based on this premise, the current study showed the activation of SIRT1 and AMPK/mTOR in skeletal muscle of ALA-treated OLETF rats. Sirtuin activation by ALA, however, requires further investigation to decipher its role in negatively or positively regulating skeletal muscle mass.

## Conclusions

ALA-induced skeletal muscle hypertrophy is a complex process involving many signal transduction pathways. Our results suggest a potential role for ALA in maintaining skeletal muscle mass by signal transduction through the AMPK/SIRT1/PGC-1α pathway in response to catabolic stress and through the AKT/mTOR/p70S6K pathway in response to anabolic stress.

## Abbreviations

ALA: Alpha-lipoic acid
AMPK: AMP-activated protein kinase
Atrogin-1: Atrogin-1/Fbx32
DHLA: Dihydrolipoic acid
FOXO1: Forkhead box O1
GADPH: Glyceraldehyde 3-phosphate dehydrogenase
IRS-1: Insulin receptor substrate-1
mTOR: Mammalian target of rapamycin
MuRF1: Muscle-specific- RING-finger protein 1
MyoD: Myogenin determination gene D
p38 MAPK: p38 mitogen-activated protein kinase
PGC-1α: peN Moxisome proliferator-activated receptor γ coactivator 1α
SIRT1: Sirtuin 1
TRF: Tocotrienol-rich fraction
